# Microbiome Yarns: human milk oligosaccharides, *Bifidobacterium* and immunopowergames^1^,^2^,^3^,^4^

**DOI:** 10.1111/1751-7915.13208

**Published:** 2018-02-23

**Authors:** Kenneth Timmis, Franziska Jebok

**Affiliations:** ^1^ Institute of Microbiology Technical University Braunschweig Braunschweig Germany; ^2^ Institute for Educational Science University of Freiburg Freiburg im Breisgau Germany

## ***Friday evening, 5.35 pm, in the main bar of the Bulls and Bears in the City (London), a favourite watering hole of Financial Masters of the Universe*** 5 ***and would‐be FMUs:***

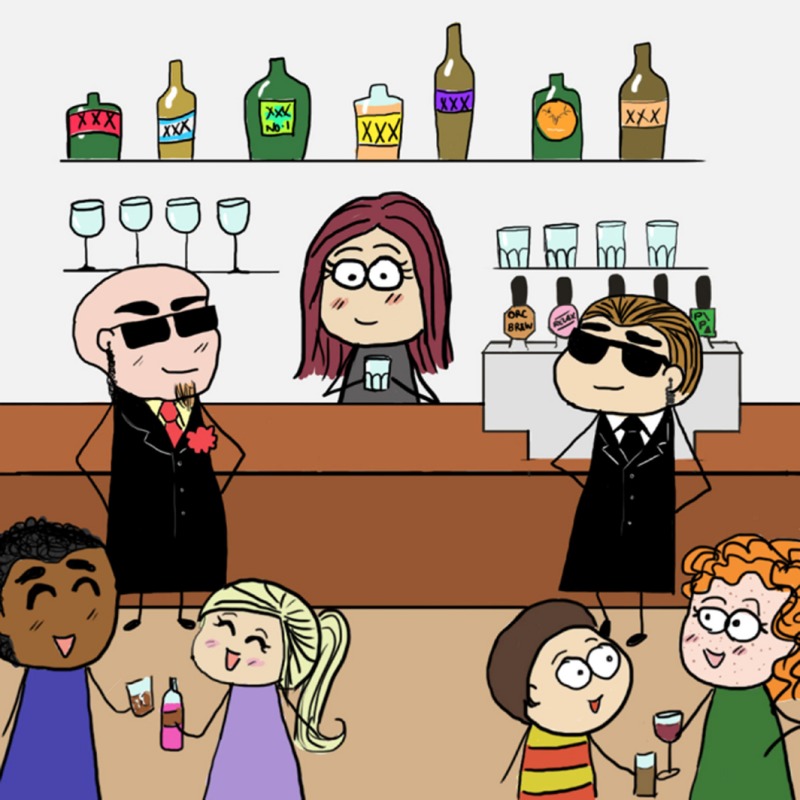

*Bondage, nonchalantly leaning against the bar*: Hi, I guess that the red carnation you are sporting indicates that you are the gentleman from *Menacyn*. Bondage, at your service!

*von Spectre*: My goodness, public schoolboys are indeed forward!

*Bondage*: Very droll! My name is Bondage, James Bondage.

*von Spectre*: Sorry, unable to resist! Good evening James, my name is Mamba von Spectre, but you can call me Mam. Shall we sit in that inconspicuous corner over there where everyone can see us but, with this happy hour noise, no‐one can hear us. Let us have a drink – my friends checked this place out in advance and it seems to be reasonably safe.

*Bondage*: Sure – let's have a Jäger‐Train^6^ between us; then we can be reasonably sure that our drink does not contain anything untoward.

*von Spectre*: Good idea. Miss! We'd like a Jäger‐Train– a 10‐er should initially suffice.

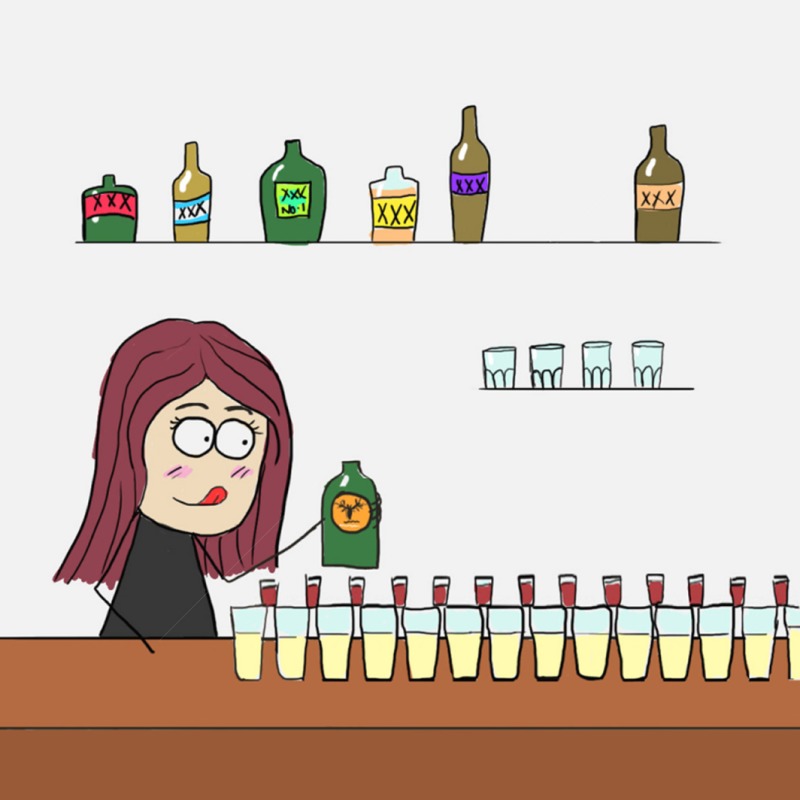

*A few minutes later, after chairs around the corner table are re‐, and re‐, and re‐arranged to provide both with adequate lines of sight to the multiple entrances and other doors*:

*Bondage*: So Mam… what does *Menacyn* have in mind this time that requires the attention of Her Majesty's Secret Service?

*von Spectre*: Ah, James – you always get straight to the point, unlike the fetching and ever‐so diplomatic Miss Geld‐Groschen…… HM Secret Service, did you say? MIH57^7^ is more like HM *Unsecret* Service, after our friends at *Wackidribbles* did their public‐spirited work.

Ok, just a bit of background to put you in the picture. As you are almost certainly aware, recent research has shown that human milk contains a remarkable number of a type of compound called human milk oligosaccharides, or HMOs, which, unexpectedly, do not nourish the baby, but instead serve as food for intestinal microbes with the moniker *Bifidobacterium*, or Bif for short^8^. Breast feeding thus provides a continual selective advantage for Bif, which has otherwise to compete with other bugs that ordinarily colonise the infant intestine.

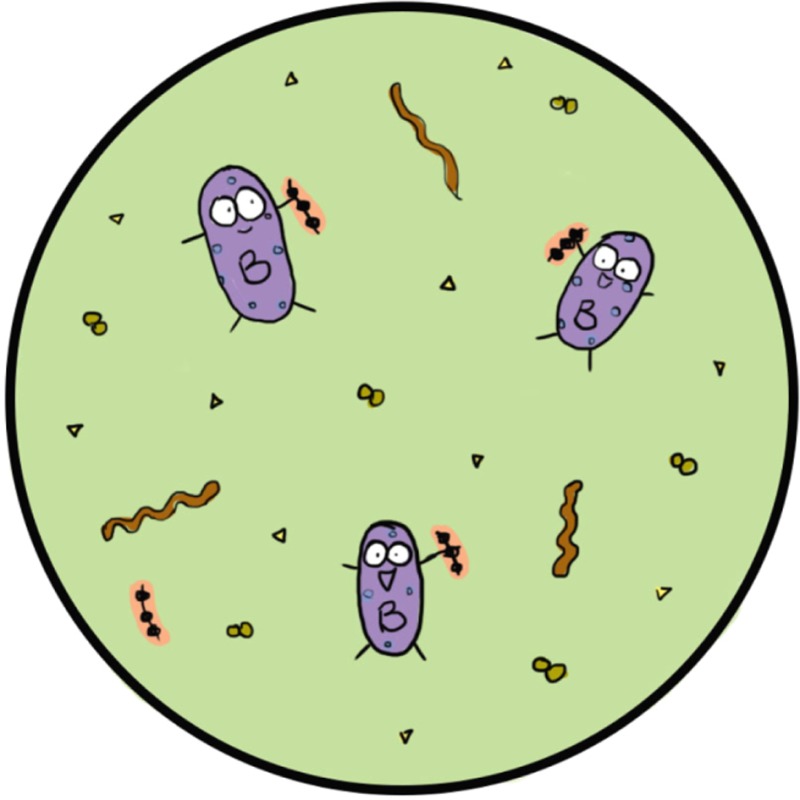

*And the neat thing is*: Bif, unlike these other bugs, orchestrates correct development of the immune system, which is essential for optimal protection against infections and development of allergies^8^. Apparently, Bif is a very hairy bug, having appendages called *tight adherence pili* and *sortase‐dependent pili*, and special polymers of sugars, that establish intimate contacts with the infant intestinal surfaces and mediate the bacterium:host dialogues, that are key to correct development of the immune and inflammatory systems. Cow's milk, though nutritious for infants, does not select for Bif, and the other bugs that colonise the infant intestine are not nearly as effective at building an effective immune system.

*Thus*: without Bif in infancy, people are less effective at handling infections and are much more prone to develop allergies. Are you following me?

*Bondage*: Of course – this is common knowledge.

*von Spectre*: Good! What is not common knowledge is that *Menacyn* scientists have developed an anti‐Bif virus cocktail that kills all known bifidobacteria, except one, a designer Bif that *Menacyn* researchers have created and named *Menacynbif*. *Menacynbif* can only grow when provided with a secret baby formula we have developed in parallel, and called *Bifgro*. We have packaged the phage cocktail in a microcapsule that ensures virus survival under all the different conditions that might be encountered in the various routes we plan for delivery, but that dissolves in the intestine to release the virus.

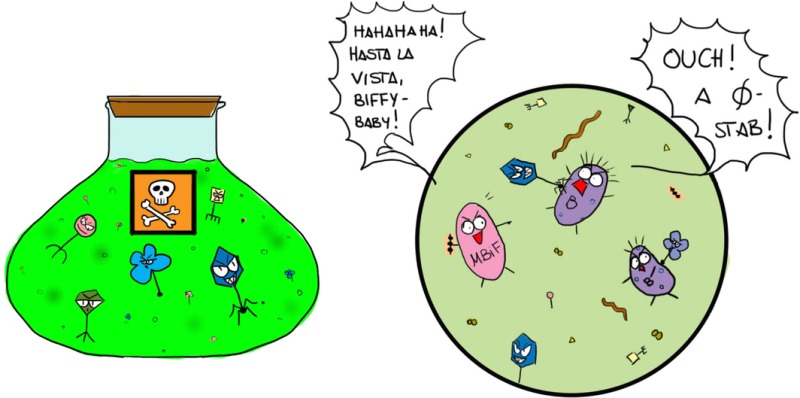

Without Bif, the world will become more susceptible to mild common infections, like flu, and nuisance allergies. Furthermore, *Menacyn* scientists have developed a powerful new synthetic allergen, called *Hisrallergen*, which provokes a histamine storm, that cannot be relieved with anti‐histamines in people deprived of Bif‐orchestrated immune development, and which causes non‐stop sneezing the whole day long. *Hisrallergen*, or *HAG* for short, is readily distributed by amateur drones. The resulting diminished visual acuity and mental attention, and the messing up of keyboards downstream of noses, means that anyone using a computer makes constant errors: imagine what this will, for example, do to the daily business of the world's financial centres!

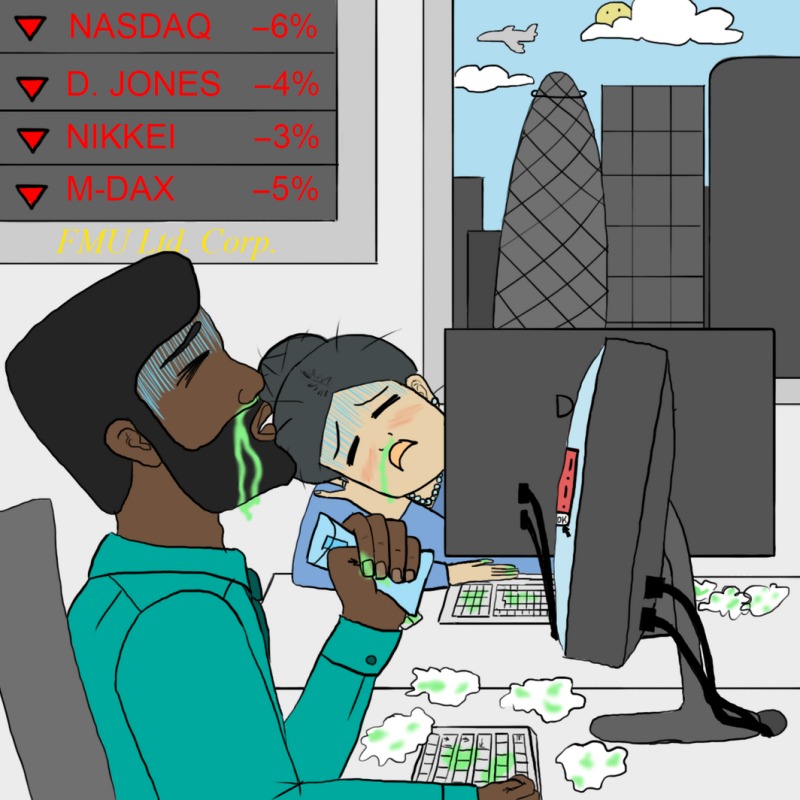

*Bondage*: Interesting… and I daresay that *Menacyn* has done all this for a reason that you would like to communicate to HM Government?

*von Spectre*: Exactly; that is the purpose of this evening's *tête‐a tête*, or Jäger‐Train… shall we order another? Miss: same again, please!

As I was saying, we have the wherewithal to eliminate Bif in countries we target with our anti‐Bif viruses, and thereby create health and economic havoc. Additionally, we have *Menacynbif*, which is not affected by the anti‐Bif viruses and orchestrates correct immune development. We now propose to offer HMGov access to *Menacynbif* for the entire population of the UK and HM Overseas Territories in return for the totality of its currency reserves, including the rather pathetic amount of gold remaining after the catastrophic fire sale^9^ in the late 90s to early 00s. Since *Menacynbif* requires certain secret nutrients in *Bifgro*, and dies without them, it will also be necessary to enter into an irrevocable 100‐year contract to purchase a national supply of *Bifgro* annually. The alternative is health and economic chaos for HM realm that would ensue which, believe me – we have done the economic evaluation^10^ calculations – would greatly exceed the cost of access to *Menacynbif*.

*Bondage*: Okay, so that is the game. Unfortunately for you, Mam, and your *Menacyn* cronies, MIH57 anticipated all this ages ago and has a neat defence in the drawer. Let me enlighten you with a little bit of the story. Some years ago, we commissioned the development of a purely synthetic, genetically‐stripped down version of Bif, called *Synbif*, which has all the desired immune stimulating properties of Bif, but is entirely resistant to all known phages. The microbiologists who developed *Synbif* – a strategic alliance between the *Lorenzo von Syntech High Security Institute for Artificial Life* in Madrid, headed by the world‐renowned Professor Vic Torde, and a secret high‐tech germ warfare defence group, reportedly located at Porton Up! – also engineered into it a couple of properties that make it super competitive, such that, when ingested, it eliminates all other Bif strains. If and when necessary, *Synbif* can be deployed anywhere in the world to eliminate *Menacynbif*, and thereby neutralise any security threat represented by *Menacynbif* for the UK and its allies. *Synbif*, like *Menacynbif*, also has specific nutritional requirements that must be delivered as a dietary supplement called *Synbifgro*, so it can itself be easily eliminated at any point after deployment, once the all‐clear is given, and Bif can be safely returned to normal duty via a probiotic.

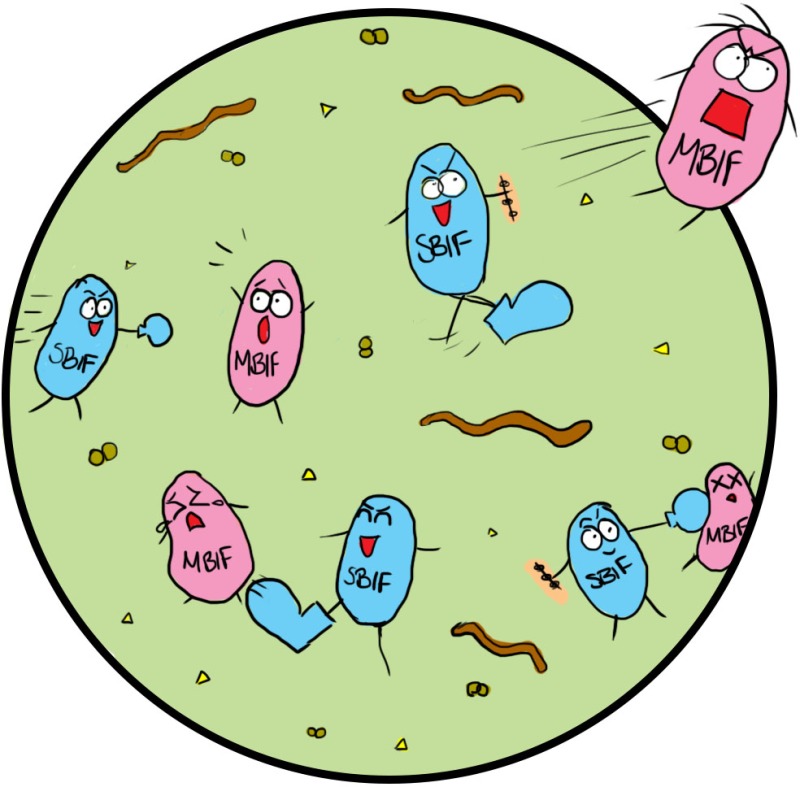

*von Spectre*: Well done James! A wonderful impromptu story! But: to have something like this, you would have had to have done some pretty serious human trials that would not have gone unnoticed, so I am rather sure that *Synbif* is a figment of your imagination.

*Bondage*: Well, Mam, you can of course believe whatever you wish, but I suppose that your *Menacyn* superiors will be at least a little nervous. And… just to make them a tad more nervous, I'll give you a little more intelligence. Three secret trials were indeed carried out: one by us on a well‐populated island off HM coast, and subsequently two of our Southern European allies each carried out a trial on one of their offshore islands. Obviously, the results have not been published, but all trials were successful.

*von Spectre*: This also does not appear credible: the famed transparency of the democratic British system would have created an uproar, so I cannot imagine HMGov wanting to run the risk of an adverse public reaction.

*Bondage*: Actually old sport, HMGov is not always as transparent as one might imagine and, since the folks on the island in question pay very little tax, and thus have little clout with the Treasury, and hence HMGov in general, MIH57 was not too concerned. So, actually I think it is *Menacyn* that is now on the back foot. It seems that we might have yet again achieved stalemate.

Miss! Please bring us that bottle of 1990 Romanée‐Conti I asked you to order for this evening: we both need to re‐educate our palates!

*Bar person*: Sorry, Sir: your favourite wine purveyor in St. James's Street ran out of the 1990 vintage on bonus day and could only provide the 1989.

*Bondage*: Dinna worry lass, the 1989 is also reasonably drinkable and will save HMGov about a grand.

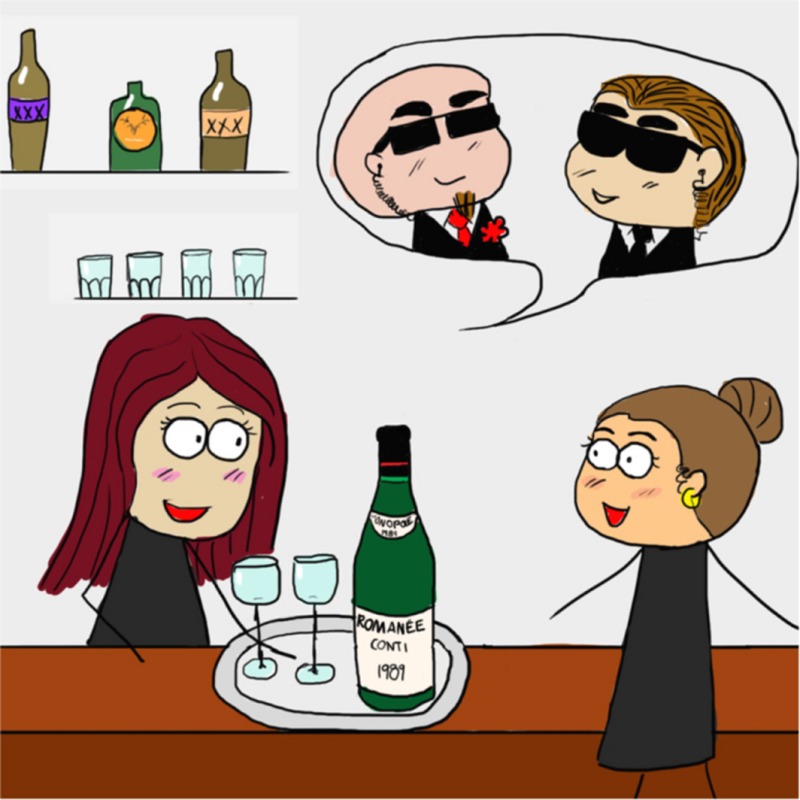

## Conflict of interest

None declared.

## Explanatory Notes

5 Masters of the Universe: see The Bonfire of the Vanities by Tom Wolfe

6
https://www.youtube.com/watch?v=6rMFNiDRrKg

7 a comprehensive reorganisation of the MI6 (Military Intelligence Section 6), following exposure of many of its operatives by Wackidribbles, and subsequent hacking attacks from 8‐year old children at a primary school in Aldershot, resulted in its breakup into multiple small, unconnected cells with MI designations reflecting favourite meals of the British military.

8 e.g. Schell, M.A., *et al*. (2002) The genome sequence of *Bifidobacterium longum* reflects its adaptation to the human gastrointestinal tract. *Proc Natl Acad Sci USA* **99**: 14422–14427; Underwood, M.A., *et al*. (2015) *Bifidobacterium longum* subspecies *infantis*: champion colonizer of the infant gut. *Pediatr Res* **77**: 229–235; Kirjavainen, P.V., Arvola, T., Salminen, S.J., and Isolauri, E. (2002) Aberrant composition of gut microbiota of allergic infants: a target of bifidobacterial therapy at weaning? *Gut* **51**: 51–55; Ventura, M., O'Flaherty, S., Claesson, M.J., Turroni, F., Klaenhammer, T.R., *et al*. (2009) Genome‐scale analyses of health‐promoting bacteria: probiogenomics. *Nat Rev Microbiol* **7**: 61–71; O'Connell Motherway, M., Zomer, A., Leahy, S.C., Reunanen, J., Bottacini, F., *et al*. (2011) Functional genome analysis of *Bifidobacterium breve* UCC2003 reveals type IVb tight adherence (Tad) pili as an essential and conserved host‐colonization factor. *Proc Natl Acad Sci U S A* **108**: 11217–11222; Fanning, S., Hall, L.J., and van Sinderen, D. (2012) *Bifidobacterium breve* UCC2003 surface exopolysaccharide production is a beneficial trait mediating commensal‐host interaction through immune modulation and pathogen protection. *Gut Microbes* **3**: 420–425; Wickramasinghe, S., Pacheco, A.R., Lemay, D.G., and Mills, D.A. (2015) Bifidobacteria grown on human milk oligosaccharides downregulate the expression of inflammation‐related genes in Caco‐2 cells. *BMC Microbiol* **15**: 172; Milani, C., Mangifesta, M., Mancabelli, L., Lugli, G.A., Mancino, W, *et al*. (2017) The sortase‐dependent fimbriome of the genus *Bifidobacterium*: extracellular structures with potential to modulate microbe‐host dialogue. *Appl Environ Microbiol* **83**: e01295‐17

9
https://en.wikipedia.org/wiki/Sale_of_UK_gold_reserves,_1999-2002

10 Drummond, M.F., Sculpher, M.J., Claxton, K., Stoddart, G.L., and Torrance, G.W. (2015) Methods for the Economic Evaluation of Health Care Programmes. 4th ed: Oxford: Oxford University Press.

